# Reading first or smelling first? Effects of presentation order on odor identification

**DOI:** 10.3758/s13414-014-0811-3

**Published:** 2014-12-18

**Authors:** A. Sorokowska, E. Albrecht, T. Hummel

**Affiliations:** 1 Smell & Taste Clinic, Department of Otorhinolaryngology, Technische Universität Dresden, Fetscherstrasse 74, Dresden, 01307 Germany; 2 Institute of Psychology, University of Wroclaw, Wroclaw, Poland

**Keywords:** Olfactory perception, Sniffin’ Sticks, Odor identification, Cued identification

## Abstract

Verbal labels are potent manipulators for olfactory perception, and verbal descriptors used in a cued olfactory identification test will influence the testing results. The main aim of the present study was to test whether the order of presentation of the odorants and the corresponding set of labels (verbal descriptors with or without pictures) would influence the results of a psychophysical odor identification test in 100 normosmic subjects (49 women and 51 men) and 100 patients with olfactory dysfunction (61 women and 39 men). Additionally, we investigated whether the scores would be different between subjects identifying odors from a list of verbal descriptors and subjects using both pictures and verbal descriptors. The subjects were examined with the extended, 32-item “Sniffin’ Sticks” identification test. We found that the scores of normosmic subjects were significantly higher when the subjects were presented with label options prior to smelling, whereas for patients the scores in the two conditions did not differ. Moreover, in both groups the scores were not significantly different when the subjects were presented either with verbal descriptors only or with verbal descriptors and pictures. Our findings seem to be of importance not only to research involving psychophysical olfactory identification tests or in a clinical context, but also to further experiments investigating human olfaction and cognition.

Odor identification is usually defined as the ability to name an odor. It is a higher-order olfactory function involving both sensation and cognition—in steps of odor detection, accurate recognition, and finding an appropriate verbal label in semantic memory (Doty, 2005; Murphy, Nordin, & Acosta, 1997)—and identification tests’ results are often related to the cognitive performance of subjects (Hedner, Larsson, Arnold, Zucco, & Hummel, 2010; Westervelt, Ruffolo, & Tremont, 2005). Given the cognitive component of the identification abilities, it is not surprising that identification performance is much lower in a free than in a cued identification task—that is, the selection of an appropriate odor label from a list with a few verbal descriptors (Distel & Hudson, 2001). In an uncued identification task, naming performance rarely exceeds 50 % (Cain, 1979; Distel & Hudson, 2001; Engen, 1987; Jönsson & Olsson, 2003). Subjects are often able to recognize an odor as familiar and pertaining to some general category, but they are still unable to find a correct verbal label. This is described as the “tip-of-the-nose phenomenon” (Lawless & Engen, 1977).

Since cued identification is considerably easier and less cognitively demanding than free identification (e.g., Cain, de Wijk, Lulejian, Schiet, & See, 1998; Engen, 1987), the vast majority of the existing psychophysical olfactory identification tests are based on a multiple forced choice task; typically, odor identification testing involves the identification of an odorant from a list of a few verbal descriptors or pictures (e.g., UPSIT; Doty, Shaman, Kimmelman, & Dann, 1984). The Sniffin’ Sticks test (Hummel, Kobal, Gudziol, & Mackay-Sim, 2007; Hummel, Sekinger, Wolf, Pauli, & Kobal, 1997; Kobal et al., 1996) is one of the most popular methods of this type. The original version of the test consists of three subtests enabling the diagnosis of different aspects of olfactory function: tests for odor threshold (OT), odor discrimination (OD), and odor identification (OI). Each of these subtests contains 16 items. The “Sniffin’ Sticks” have been used in hundreds of scientific studies and are widely used for clinical purposes. In addition to the “classical,” 16-item version of the identification and discrimination subtests, extended, 32-item versions of these two tests also exist (Haehner et al., 2009). They were developed in order to create more precise tools that would enable researchers to perform repeated, longitudinal testing of individual olfactory subfunctions.

Some studies have shown that the verbal labels applied in an olfactory identification test might influence the testing results. First of all, the distinctiveness of the distractors and the target odor might affect the identification score (Engen, 1987), with more contrasted distractors improving the test results (Gudziol & Hummel, 2009), and higher numbers of labels decreasing the subject’s performance (Negoias, Troeger, Rombaux, Halewyck, & Hummel, 2010). Other than that, surprisingly little is known about interactions between olfactory stimuli and verbal information in psychophysical olfactory identification tests, including the “Sniffin’ Sticks” test. Yet verbal labels are potent manipulators for olfactory perception (Herz, 2000), and they often provide a frame of reference for olfactory stimuli (Herz & von Clef, 2001). Added to this, verbal information might strongly influence the perception of an odor’s attributes—for example, hedonic ratings of an odorant (de Araujo, Rolls, Velazco, Margot, & Cayeux, 2005; Herz, 2003; Herz & von Clef, 2001), or even of clean air (de Araujo et al., 2005; Herz & von Clef, 2001; Slosson, 1899). Congruent visual stimuli can also magnify an odor’s intensity and pleasantness (Sakai, Imada, Saito, Kobayakawa, & Deguchi, 2005; Seo et al. 2010), and olfactory detection can be faster and more accurate when odors appear with semantically congruent visual cues (Gottfried & Dolan, 2003).

Generally, it is not clear in which order odorants and the corresponding set of verbal descriptors/pictures in the Sniffin’ Sticks (or any other) identification test should be presented—should a subject smell an odor first and then read a list of descriptors, or should reading the list of possible answers be followed by odor presentation? The difference between these two methods might seem subtle, but in fact the first version of the procedure (i.e., first smelling, then reading) is much more similar to noncued identification than is the second version (i.e., first reading, then smelling). Furthermore, in the discussion of their results, Herz and von Clef (2001) suggested that when people smell an odor without first being provided with verbal label, even when they are not actually asked to identify the odor, they still try to generate some kind of label after smelling. Possibly an association that one might have with a certain odor is not always fully congruent with the options provided in an identification task, which might lead to more errors when choosing a label from a list of descriptors, than when the options are read prior to smelling. Previous studies on the Sniffin’ Sticks identification test have not explored this issue, and we aimed to resolve this problem in the present study.

In addition to the examination of the effect of presentation of the odor stimulus and the corresponding labels, in the present study we investigated one more issue related to the extended identification test. In the “original” version of the identification test, the scores of subjects identifying odors from a list of verbal descriptors were not significantly different from those of subjects using both pictures and verbal descriptors (Hummel et al., 1997). However, this issue has not been tested in the extended version of the test (Haehner et al., 2009).

## Method

### Materials and methods

Investigations were performed according to the Guidelines for Biomedical Studies Involving Human Subjects (Helsinki Declaration). The protocol was approved by the Ethics Committee of the Medical Faculty of TU Dresden (Application No. 156052012). All subjects provided written informed consent prior to their inclusion in the study.

### Subjects

The study comprised 100 normosmic, healthy people (49 women, 51 men) 22–70 years of age (*M* = 36.3, *SD* = 15.9), and 100 patients with olfactory loss (61 women, 39 men) 24–85 years of age (*M* = 59.2, *SD* = 13.4). The subjects underwent diagnostic evaluation; they received a detailed otorhinolaryngological investigation including a medical interview and nasal endoscopy. We did not obtain information on the causes of the olfactory loss of the patients. However, on the basis of the overall frequencies of causes of olfactory loss at the Smell and Taste Clinic in the years 2012 to present (total: *N* = 1,572 cases), one can assume that the distribution was approximately as follows: congenital 1 %, idiopathic 21 %, trauma 15 %, postviral 37 %, sinunasal 20 %, and other 5 %. Additionally, according to the normative values for the extended identification test (Sorokowska, Albrecht, Hähner, & Hummel, under review), in our sample 41 % of the subjects were hyposmic, and 39 % were functionally anosmic.

### Procedure

The subjects were examined with the extended, 32-item “Sniffin’ Sticks” identification test (cf. Haehner et al., 2009). Subjects identified the odors by selecting the correct odorant name from a list of four descriptors. In the data analysis, we used three different test scores for each subject: (1) the first 16 items of the task (i.e., the “original” Sniffin’ Sticks; score 0–16), (2) the 16 new items of the task (i.e., the “new” test; score 0–16), and (3) the total for all 32 items (i.e., the full, extended version of the tasks; score 0–32).

**Fig. 1:**
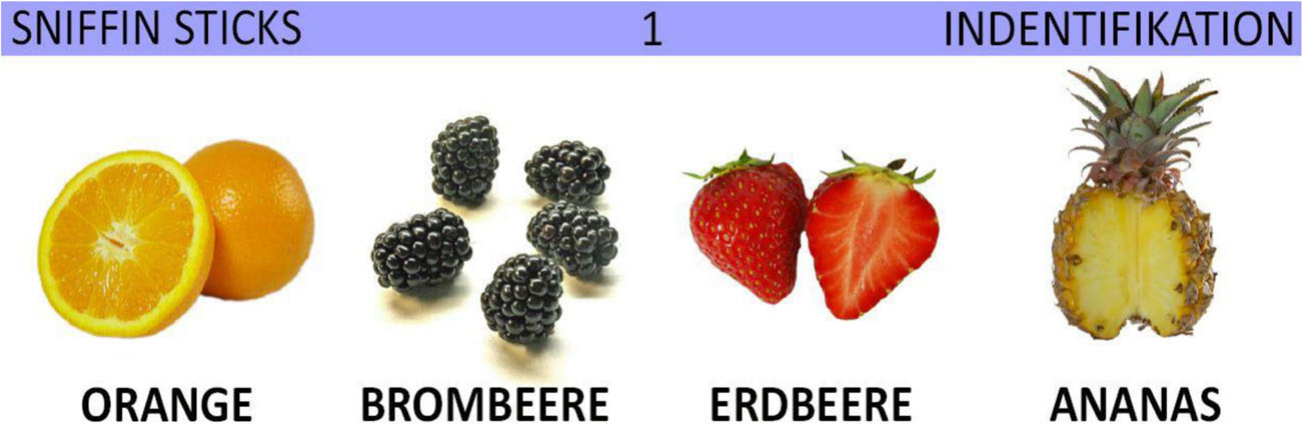
Example of a Sniffin’ Sticks identification test answer sheet combining pictures and verbal labels

Half of the subjects in each group (patients/normosmic subjects) completed the test with verbal labels only, and half of the subjects were presented with the verbal labels and with additional pictures representing the target odors and distractors (see Fig. 1).

Moreover, half of the subjects (both normosmic people and patients) were assigned to a “reading-first” condition, and half of the subjects were assigned to a “smelling-first” condition. In the reading-first condition, the subjects read four provided options (the label of a target odor and three distractors), then they smelled an odor, and afterward they selected an answer (the response alternatives were not removed during odor presentation; subjects could read the options again if they wished to do so before providing an answer, but they did not smell the odorant again). In the smelling-first condition, the subject smelled an odor first, read provided labels afterward, and then selected an answer. Assignment to the groups was random—the subjects were assigned to the testing schedule in the order of their appearance at the Smell and Taste clinic.

Statistical analyses were performed by means of Statistica Version 10 (StatSoft, Inc.; www.statsoft.com, Tulsa, OK). The scores obtained in the various conditions were compared through independent-sample *t* tests and a two-way analysis of variance. The level of significance was set at .05. Correlational analyses were performed using Pearson’s method.

## Results

### Comparison between healthy subjects and patients with olfactory dysfunction

The scores of healthy subjects were significantly higher than those of patients with olfactory dysfunction in the “original” 16-item test [healthy subjects: *M* = 13.25, *SD* = 1.73; patients: *M* = 8.62, *SD* = 4.16; *t*(198) = 10.28, *p* < .001], the “new” 16-item test [healthy subjects: *M* = 13.36, *SD* = 2.12; patients: *M* = 8.94, *SD* = 3.68; *t*(198) = 10.40, *p* < .001], and the full, 32-item test [healthy subjects: *M* = 26.61, *SD* = 3.34; patients: *M* = 17.56, *SD* = 7.43; *t*(198) = 11.11, *p* < .001].

### Order of presentation

We observed significant differences between the reading-first and smelling-first conditions in all three tests (“original,” “new,” and “extended”), as indicated by independent-sample *t* tests. In the case of patients with olfactory dysfunction, the differences were nonsignificant (all results are presented in Table 1). When the labels were read before the odor was smelled, the percentage of correct identifications of all odorants was, on average, 6.44 % higher.

**Table 1:** Differences in scores of normosmic subjects and patients in “reading-first” and “smelling-first” conditions in three versions of the Sniffin’ Sticks identification test (“original,” “new,” and “extended”), as indicated by independent-sample *t* tests

	Reading First		Smelling First		Difference in Average Score (Absolute Value)	*t*-Test Value	*p* Value
	*M*	*SD*	*M*	*SD*			
Normosmic Subjects	*n* = 50		*n* = 50				
“Original” 16-item test	13.70	1.13	12.80	2.10	0.90	2.68	.009
“New” 16-item test	13.94	1.61	12.78	2.42	1.16	2.82	.006
Extended 32-item test	27.64	2.18	25.58	3.94	2.06	3.23	.002
Patients	*n* = 54		*n* = 46				
“Original” 16-item test	8.96	3.88	8.22	4.47	0.74	0.89	.37
“New” 16-item test	9.04	3.86	8.83	3.50	0.21	0.28	.78
Extended 32-item test	18.00	7.26	17.04	7.67	0.96	0.64	.52

### Verbal labels versus pictures

Table 2 presents differences in the scores of normosmic subjects and of patients who received answer sheets either with only verbal labels or with verbal labels and pictures. The differences were nonsignificant for both normosmic subjects and patients in all tests (see the *t*-test results in Table 2). Additionally, to test the influence of age on the performance of normosmic subjects in these two conditions, we performed a two-way analysis of variance with the factors Age (younger subjects: 56 people 22–35 years old vs. older subjects: 44 people 36–70 years old) and Condition (verbal labels only vs. verbal labels and pictures). Only the main effect of age was significant [*F*(1, 96) = 13.34, *p* < .001, *ƞ* ^2^ = .12]. Neither the main effect of condition [*F*(1, 96) = 0.59, *p* = .44, *ƞ* ^2^ = .01] nor the interactive Age × Condition effect [*F*(1, 96) = 0.57, *p* = .45, *ƞ* ^2^ = .01] was significant.

**Table 2:** Differences in scores of normosmic subjects and patients in “verbal labels only” and “verbal labels and pictures” conditions in three versions of the Sniffin’ Sticks identification test (“original,” “new,” and “extended”), as indicated by independent-sample *t* tests

	Verbal Labels and Pictures		Verbal Labels Only		Difference in Average Scores (Absolute Value)	*t*-Test Value	*p* Value
	*M*	*SD*	*M*	*SD*			
Normosmic Subjects	*n* = 50		*n* = 50				
“Original” 16-item test	13.10	1.96	13.40	1.47	.30	–0.87	.39
“New” 16-item test	13.34	2.40	13.38	1.83	.04	–0.09	.93
Extended 32-item test	26.44	3.94	26.78	2.63	.34	–0.51	.61
Patients	*n* = 49		*n* = 51				
“Original” 16-item test	8.61	4.30	8.63	4.06	.02	–0.02	.99
“New” 16-item test	8.65	3.71	9.22	3.67	.57	–0.76	.45
Extended 32-item test	17.27	7.51	17.84	7.41	.57	–0.39	.70

## Discussion

The main aim of the present study was to test whether the order of presentation of the odorants and the corresponding set of labels (verbal descriptors/pictures) influences the results of a psychophysical odor identification test in normosmic subjects and/or patients with olfactory dysfunction. We found that the scores of normosmic subjects were significantly higher when they were presented with label options prior to smelling, whereas for patients the scores in the two conditions did not differ. Moreover, in both groups the scores were not significantly different when the subjects were presented with verbal descriptors only or with verbal descriptors and pictures.

Previous studies have shown that the perception of an odor can be significantly influenced by a provided label (de Araujo et al., 2005; Herz, 2003; Herz & von Clef, 2001). In our study, we demonstrated that healthy, normosmic subjects performed better in an olfactory identification task when they could first read a list of potential odor labels. This finding is particularly interesting, since it shows that the involuntary and intuitive identification attempts performed prior to learning the possible answer options might somehow distort our olfactory perception. A possible explanation of our result is verbal priming in odor perception—the so-called “first-label effect” (Herz & von Clef, 2001). Since the connotation of a label given to an odorant influences further responses to this fragrance, the cued identification—performed after reading the possible odor labels—is somehow “guided” by these labels. Conversely, an incorrect first association that one might have with a certain odor might distract from the further choice of a label from a list of descriptors.

Interestingly, the order of presentation of the verbal labels and smells did not influence the scores in patients, although that effect was very clearly seen in healthy controls. Why did the order of presentation have little or no effect in this group? We might present a few explanations; however, at this stage of research all of the options remain purely hypothetical. First, it is possible that since the data in patients are noisier—the standard deviations are higher, and generally the results show more variance (see, e.g., Haehner et al., 2009)—this could suppress all of the possible effects of stimulus presentation. Additionally, since there is rather high variability in the patients’ scores, and since the patients have considerably lower scores than do normosmic subjects, a floor effect might also, at least partly, explain the lack of a difference between the compared conditions in the patients group. Second, the decreased olfactory sensitivity of patients obviously makes their everyday “smelling experiences” less pleasant and more vague, simply because their sense of smell is weaker. Moreover, participation in olfactory tests is more demanding and more difficult for patients than for normosmic people. Thus, because patients might already be tired and frustrated when sniffing the odors, they may give up searching for specific odor qualities before reading the labels (see Doty, Genow, & Hummel, 1998), and their expectations would not interfere with the answer options provided.

Our study showed also that pictures, presented together with verbal labels of the odorants, are of no advantage, either to patients with olfactory dysfunctions or to normosmic subjects (similar to what Hummel et al., 1997, observed). Also, the effects were not different in any age group, which is consistent with previous findings regarding the “original” Sniffin’ Sticks (Sorokowska et al., 2014). Generally, it seems that adding pictures to the answer sheets does not improve performance in the identification test. In theory, pictures may even decrease the scores. First, they might distract the subjects’ attention, which could negatively influence their performance because olfactory identification is a demanding cognitive task (Westervelt, Bruce, Coon, & Tremont, 2008). Second, in some tests pictures could provide additional, and possibly even incongruent, information with regard to the verbal label, which could then produce a conflict when selecting the proper answer.

Our findings seem to be of particular importance not only for research involving psychophysical olfactory identification tests, but also for further experiments investigating human olfaction and cognition. In a clinical context, our results mean that patients/subjects should always read the labels first, before the odor is presented. Considering that the effects of label presentation were more pronounced in healthy controls than in patients, this procedure should allow for better discrimination between controls and people with olfactory loss. Additionally, our study showed that in a clinical context, adding pictures to verbal labels is not helpful during odor identification tests in adults.
